# Childhood socioeconomic position and healthy ageing: results from five harmonised cohort studies in the ATHLOS consortium

**DOI:** 10.1136/bmjph-2024-001590

**Published:** 2025-02-26

**Authors:** Yu-Tzu Wu, Sam Gnanapragasam, Albert Sanchez-Niubo, Muhammad Zakir Hossin, Ilona Grünberger, Seppo Koskinen, Rachel Cooper, Matthew Prina

**Affiliations:** 1 Population Health Sciences Institute, Faculty of Medical Sciences, Newcastle University, Newcastle upon Tyne, UK; 2 Department of Health Service and Population Research, Institute of Psychiatry, Psychology & Neuroscience, King’s College London, London, UK; 3 Department of Social Psychology and Quantitative Psychology, University of Barcelona, Barcelona, Spain; 4 Centro de Investigación Biomédica en Red de Salud Mental (CIBERSAM), Instituto de Salud Carlos III, Madrid, Spain; 5 Research, Innovation and Teaching Unit, Parc Sanitari Sant Joan de Déu, Sant Boi de Llobregat, Spain; 6 Department of Medicine Solna, Clinical Epidemiology Division, Karolinska Institute, Stockholm, Sweden; 7 School of Population and Public Health, The University of British Columbia, Vancouver, British Columbia, Canada; 8 Department of Public Health Sciences, Stockholm University, Stockholm, Sweden; 9 Department of Public Health and Welfare, Finnish Institute for Health and Welfare, Helsinki, Finland; 10 NIHR Newcastle Biomedical Research Centre, Northumberland Tyne and Wear NHS Foundation Trust, Newcastle upon Tyne, UK; 11 AGE Research Group, Translational and Clinical Research Institute, Faculty of Medical Sciences, Newcastle University, Newcastle upon Tyne, UK

**Keywords:** Epidemiology, Age Factors, Sociodemographic Factors

## Abstract

**Introduction:**

Childhood socioeconomic position (SEP) has been identified as a key determinant of health. However, earlier literature is largely from high-income countries and provides limited evidence on the prolonging impacts of childhood disadvantage on healthy ageing across diverse settings and populations. The aim of this study is to investigate the associations between childhood SEP and healthy ageing across multiple countries and the mediation effects of adult SEP, individual education and wealth, on these associations.

**Methods:**

Using the harmonised dataset of five cohort studies in the Ageing Trajectories of Health-Longitudinal Opportunities and Synergies (ATHLOS) project, this study was based on 57 956 people aged ≥50 years (women: 53.3%) living in China, Finland, UK, Poland, South Africa and Mexico. The associations between childhood SEP (parental education and occupation) and healthy ageing scores were examined using linear regression modelling. Causal mediation analysis was carried out to estimate the percentage of indirect effects via adult SEP (individual education and wealth).

**Results:**

Higher levels of childhood SEP were associated with higher healthy ageing scores by up to five points and similar patterns were observed across populations from different countries. The associations were mediated by adult SEP and the range of mediation effects was between 21% and 78%.

**Conclusions:**

This study found childhood SEP was associated with poor health in later life across high-income, middle-income and low-income countries. Addressing socioeconomic disadvantage, such as improving education attainment, may moderate the impacts of adversity in early life and support health and functioning in later life.

**WHAT IS ALREADY KNOWN ON THIS TOPIC**

- The relationship between childhood socioeconomic position (SEP), physical and cognitive health conditions in later life has been reported in several epidemiological studies. However, most of these studies were carried out in high-income countries and provided limited evidence for older people living in diverse settings across the world. In addition, the existing studies mainly focused on specific diseases, syndromes and/or health conditions. There is a need to incorporate the recent WHO consensus concept of healthy ageing, using a comprehensive measure including multiple dimensions of health and functioning.

**WHAT THIS STUDY ADDS**

- Based on a harmonised dataset of nearly 58 000 older people (aged 50 or above) in six countries, we used comparable measures to investigate the associations between childhood SEP (parental education and occupation) and healthy ageing and the mediation effects of adult SEP (education and wealth) on these associations. We found positive associations between childhood SEP and healthy ageing scores in older people across different countries. The associations were attributed to adult SEP by 21%–78% across different populations.

**HOW THIS STUDY MIGHT AFFECT RESEARCH, PRACTICE OR POLICY**

- Our findings highlight opportunities to ameliorate the negative sequelae of childhood socioeconomic disadvantage by maximising education attainment and financial security in adulthood. This provides empirical evidence to inform policies and practices addressing socioeconomic determinants of healthy ageing and mitigate health inequalities in older age. Future research should adopt a life-course approach and investigate a variety of mediators (eg, cultural, societal and environmental factors) to provide a nuanced understanding of global health and ageing.

## Introduction

 Ageing is associated with increased risk of non-communicable diseases, frailty and cognitive and functioning decline.^1^ The increasing number of older population and related health conditions carry significant economic and social care considerations and burdens.^2^ To address issues related to population ageing, the concept of ‘healthy ageing’ has been highlighted in research, policy and practice.^3 4^ In 2015, the WHO provided a comprehensive definition of healthy ageing as the ‘process of developing and maintaining functional ability that enables well-being in older age’.^5^ This definition highlights functional ability, the capabilities that enable people to be and do what they have reason to value,^6^ and further considers two separate components: intrinsic capacity and environments.^7^ Intrinsic capacity relates to an individual’s mental and physical capacities. Environment is a wide-ranging concept and includes various physical and social factors and structures which restrict or promote functioning. Functional ability is the way in which an individual’s intrinsic capacity interacts with the environment. Instead of focusing on specific diseases, recent research has started to emphasise functional ability and develop operationalised measures for healthy ageing.^8^

Socioeconomic position (SEP) is the measure of an individual’s combined social and economic factors that influence their individual position within the societal structure.^9 10^ The most commonly used measures to indicate SEP, such as education, occupation, income, wealth and assets,^9^ mainly focus on economic and social resources in adulthood (ie, adult SEP). These measures may not reflect SEP in early life such as childhood disadvantage and adversity. Childhood SEP is commonly defined by parental measures (parental education and parental occupation) and household measures (household income and household conditions).^9^ These SEP indicators in childhood and adulthood have been related to health at different life stages.^11^,^13^ In particular, there is an understanding that childhood SEP has long-lasting health effects in later life. This has been described as the ‘long arm of childhood’, with childhood SEP thought to increase the risk of chronic disease and low physical functioning in later life and premature mortality.^14^

Over the last two decades, there has been growing evidence related to the relationship of childhood SEP with late-life health trajectories,^15^,^17^ cognitive status,^18^ well-being^19^ and functional status.^20^ In particular, evidence from the British birth cohort studies has identified the associations between childhood SEP and multiple physical health conditions in adulthood.^21^,^24^ However, the measure of later-life health in the context of childhood SEP has often focused on specific diseases, syndromes and/or health domains. There is a need to understand its association with contemporary and encompassing measures including multiple dimensions of health and functioning. Further, most existing studies considering the impacts of childhood SEP on later-life health are based on populations in high-income countries. This provides limited evidence for older populations living in diverse settings with varying economic development, societal structures and cultures. Given that two-thirds of older people in 2020 live in low-income and middle-income countries (LMICs),^6^ it is important to understand how SEP in childhood and adulthood can have the potential impacts on healthy ageing across diverse populations and countries.

A recent study based on the Ageing Trajectories of Health-Longitudinal Opportunities and Synergies (ATHLOS) project used the harmonised data from eight longitudinal cohort studies to investigate the potential impacts of adult SEP, measured by education and wealth, on trajectories of healthy ageing across older people in multiple countries. Education and wealth were related to baseline healthy ageing scores but were not found to affect decline rates over time.^25^ Built on this previous study and the ATHLOS harmonised dataset,^26^ we examined the associations between childhood SEP and healthy ageing across five cohort studies from Europe and LMICs. We further investigated the mediating roles of adult SEP (education and wealth) on the pathway between childhood SEP and healthy ageing.

## Material and methods

### Study population

The ATHLOS Project is a cohort consortium funded by the European Union Horizon 2020.^16^ The aim of the project was to harmonise a wide range of sociodemographic, lifestyle, social environment, physical and psychological health factors across 17 ageing cohort studies and to investigate trajectories of healthy ageing and potential risk factors and determinants. More detailed information on the harmonisation process is provided at: https://github.com/athlosproject/athlos-project.github.io. This study focused on five cohort studies which had available data on retrospectively ascertained parental occupation and education (n=73 825): the China Health and Retirement Longitudinal Study (CHARLS),^27^ the English Longitudinal Study of Ageing (ELSA),^28^ the Health, Alcohol and Psychosocial factors in Eastern Europe Study (HAPIEE),^29^ the Health 2000–2011 Survey (H2000/11)^30^ and WHO Study on Global Ageing and Adult Health (SAGE).^31 32^ To investigate the potential impact of SEP in earlier life stages on health in later life, people aged 49 or below at the baseline were excluded from analyses as they did not meet the inclusion criteria of being of older age (n=12 742). Those with missing age and sex were removed (n=3127) as there was limited information in these participants. This left 57 956 people aged 50 or above from six countries in Europe (Finland, UK and Poland), East Asia (China), Latin America (Mexico) and Africa (South Africa).

All cohort studies were approved by the relevant local research ethics committees.

### Healthy ageing scores

The process of generating the ATHLOS healthy ageing scores is reported in the literature.^25 33^ In brief, researchers from the consortium identified 41 items related to health, physical and cognitive functioning across the 17 cohort studies and harmonised these items into binary variables (presence vs absence of impairment). More detailed information on the items is provided in online supplemental table S1, supporting information. A two-parameter item-response theory model was employed to incorporate all the items and estimate a latent trait score reflecting individual health and functioning level. To test the assumption of unidimensionality, the model fit was assessed using the root mean square error of approximation, the Comparative Fit Index and the Tucker-Lewis index. To generate a common scale across all participants in the ATHLOS harmonised dataset, the model was applied to the baseline data of 343 915 people across all countries and cohort studies in the harmonised dataset. A logistic regression framework was used to detect differences in item parameters across cohort studies and the Stocking-Lord equating approach was implemented to rescale the different parameters.^33^ The scores were normally distributed with a mean of 50 and a SD of 10 (range 0–100), with higher score indicating better healthy ageing.

### Childhood SEP

Childhood SEP was defined by two measures: parental education and parental occupation. These two measures were harmonised using survey data on father’s and mother’s occupation and education where available. The cohort participants were asked to retrospectively recall their father’s and mother’s occupation and education from a list of occupations and educational qualifications presented to them. Each of these original categories (mother’s occupation, father’s occupation, mother’s education and father’s education) in the individual cohort studies were reviewed by experts in the ATHLOS consortium and regrouped into three levels: low, middle and high. The categorisation was designed to provide comparable measures of SEP for the participants across different cohort studies. Where there was a difference between characteristics of the parents on the same measure (eg, occupation), the one with a higher level was used to generate the harmonised childhood SEP variable, indicating the best possible resources in the household. Resultantly, two measures were created for each cohort participant—an occupation-based measure (parental occupation—highest between the two parents) and an education-based measure (parental education—highest between the two parents). Apart from H2000/11, all the cohort studies had the occupation-based measure. For parental education, the information was unavailable in ELSA and HAPIEE. More detailed information is provided in online supplemental table S2, supporting information.

### Adult SEP

Adult SEP was measured by education and wealth, which were harmonised across cohort studies included in ATHLOS. Education was categorised into the three levels of education: low (up to primary education), middle (secondary) and high (tertiary). Wealth was harmonised using relevant measures for personal or household income and finance (such as property, pension or insurance). The variable was divided into quintiles within specific cohorts and the highest quintile (Q5) was the most affluent group in the cohort population. All the five cohort studies had the harmonised education variable but the wealth measure was not available in HAPIEE. More detailed information is provided in online supplemental table S3, supporting information.

### Analytical strategy

**Figure 1:**
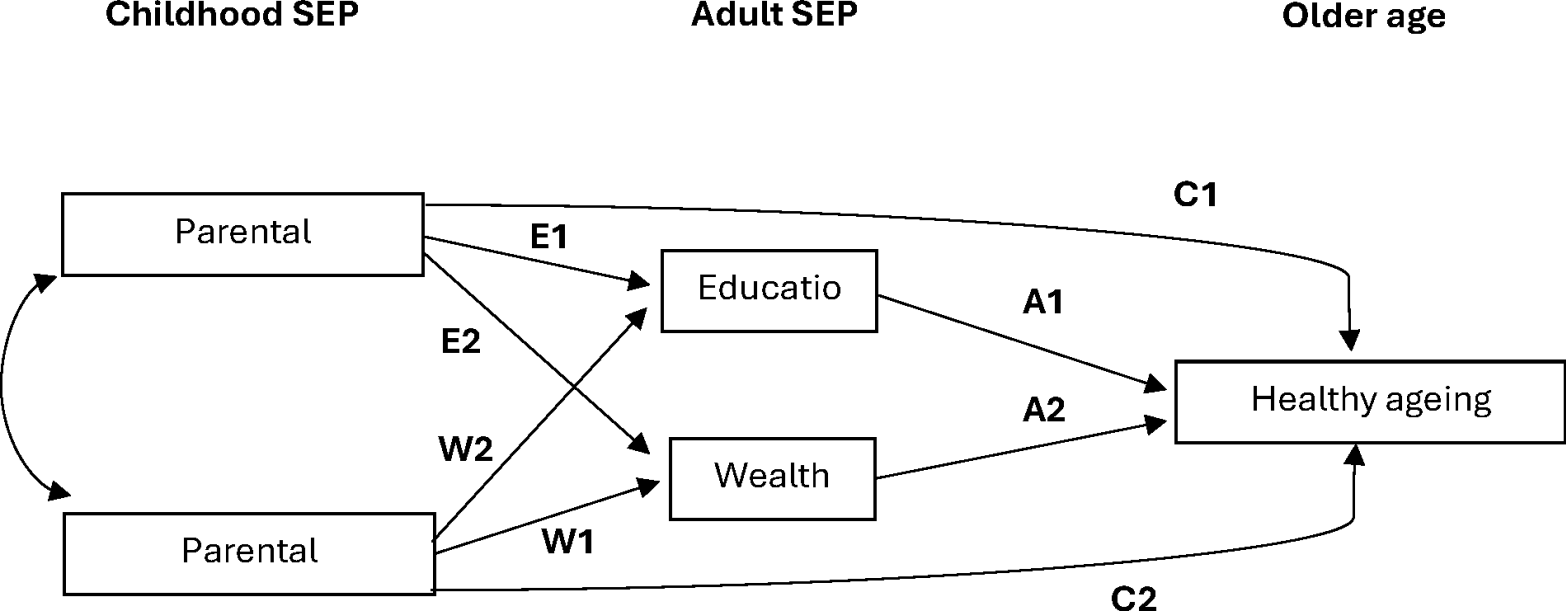
The hypothesised pathways between childhood SEP, adult SEP and healthy ageing scores. SEP, socioeconomic position.

Descriptive analyses were carried out to investigate distributions of childhood and adult SEP, healthy ageing scores, age and sex across the five cohort studies. Given that childhood and adult SEP occurred before older age, it was plausible to investigate the potential effects of childhood and adult SEP on the baseline scores for healthy ageing. To examine how the associations between childhood SEP and healthy ageing were mediated by adult SEP, three types of analytical methods were implemented:

1. *The change-in-coefficient method—for trends across the three SEP levels:* linear regression modelling was employed to investigate the associations between the two measures for childhood SEP and healthy ageing scores adjusted for age and sex (model 1). To examine whether the associations of parental education and parental occupation with healthy ageing were mediated by adult SEP, education and wealth were included in the age-adjusted and sex-adjusted models separately (models 2 and 3) and jointly (model 4). This was based on the classical change-in-coefficient approach, focusing on whether the effect sizes of childhood SEP were attenuated after adjustment. Test for trends was used to examine whether healthy ageing scores gradually increased with higher levels of childhood SEP measures and whether the trends remained similar after adjusting for adult SEP. Additional analyses were carried out to investigate the associations between childhood SEP and healthy ageing in men and women separately. Interaction terms between sex and both parental education and occupation were fitted to test whether the associations differed between men and women.
2. *Causal mediation analysis—for percentages of natural indirect effects:* this method was used to estimate the percentages of indirect effects (via adult SEP) on the associations between childhood SEP and healthy ageing.^34 35^ To reduce the complexity of the analysis, measures for childhood and adult SEP were categorised into binary groups (high/middle vs low). Based on the results of linear regression, differences in healthy ageing scores were larger between the middle and low levels of childhood SEP than between the high and middle levels. Thus, the high/middle levels were combined. The natural direct (childhood SEP) and indirect effects (via adult SEP) and the total effect (sum of natural direct and indirect effects) were estimated with adjustment for age and sex. Further adjustment was carried out to include education or wealth as confounding factors.
3. *Generalised structural equation modelling—for joint effects of adult SEP measures:* to estimate the mediating effects taking into account both adult SEP measures, generalised structural equation modelling was used to estimate the hypothesised pathways between childhood SEP, adult SEP and healthy ageing (figure 1). The relationships between childhood and adult SEP were modelled using ordinal logistic regression and their joint effects on healthy ageing scores were estimated using linear regression. This provided a full picture of all the pathways but the estimation of the indirect effects would not be straightforward due to the discrete mediators. Yet the results would show the effect sizes of the associations between childhood SEP and healthy ageing scores after accounting for all adult SEP measures, age and sex.

Given large variation across studies, all the models were carried out in individual cohorts (CHARLS, ELSA, HAPIEE, H2000/11) and the three countries included in SAGE (China, Mexico and South Africa). Due to lack of available data on parental education in ELSA and HAPIEE, the analyses focused on the occupation-based measure in these cohort studies. Since comparable information on wealth was not available in HAPIEE, only education was included in the modelling. Although meta-analysis could be used to generate a pooled estimate of all studies/countries, the results varied considerably across cohort studies and a summarised coefficient of the overall population would not be meaningful. To address missing data, multiple imputation was carried out to generate 50 imputed datasets. The imputation model was based on all variables included in the modelling (age, sex, healthy ageing scores, childhood SEP and adult SEP) and additional variables which might inform SEP and health in the ATHLOS harmonised dataset (rural/urban areas, self-rated health and country). Rubin’s rule was used to combine estimates from the imputed datasets.^36^ All analyses were carried out using Stata V.17.0.^37^

### Patient and public involvement

Patients or the public were not involved in the design, conduct or reporting of this study.

## Results

Table 1 shows descriptive information on the study populations. Among the 57 956 people aged 50 or above, nearly 47% were from two cohorts in China and 4% were from Mexico. The mean age was 63.2 years with a SD of 9.5 years. The percentage of women was 53.3% in the overall population with a range between 50.4% in CHARLS and 60.5% in SAGE—Mexico. The distributions of childhood SEP varied across cohort studies from Western Europe (ELSA, H2000/11) and other regions (CHARLS, HAPIEE, and SAGE). Among all the studies, the Finnish cohort had the highest percentage of high parental education (6.5%) and ELSA had the highest percentage of high parental occupation (22.9%).

**Table 1:** Descriptive information on the study population (n=57 956)

Study	CHARLS	ELSA	HAPIEE	H2000/11	SAGE	SAGE	SAGE
Country	China	England	Poland	Finland	China	Mexico	South Africa
Total	13 767	11 522	8747	4402	13 367	2311	3840
Age							
Mean (SD)	62.2 (8.6)	65.1 (10.3)	59.4 (5.8)	66.1 (11.8)	63.2 (9.4)	68.4 (9.5)	62.7 (9.7)
Sex							
Women	6942 (50.4)	6292 (54.6)	4424 (50.6)	2543 (57.8)	7093 (53.1)	1397 (60.5)	2203 (57.4)
Men	6825 (49.6)	5230 (45.4)	4323 (49.4)	1859 (42.2)	6274 (46.9)	914 (39.6)	1637 (42.6)
Childhood SEP							
Parental education							
Low	7381 (94.7)	NA	NA	2732 (77.1)	11 358 (89.6)	1921 (94.1)	2520 (85.9)
Middle	360 (4.6)			582 (16.4)	1115 (8.8)	56 (2.7)	317 (10.8)
High	57 (0.7)			230 (6.5)	205 (1.6)	65 (3.2)	97 (3.3)
Missing	5969			858	689	269	906
Parental occupation							
Low	6375 (84.8)	4020 (35.5)	3620 (69.3)	NA	7135 (80.0)	1332 (83.0)	1199 (44.5)
Middle	410 (5.5)	4709 (41.6)	932 (17.8)		992 (11.1)	209 (13.0)	1279 (47.4)
High	734 (9.8)	2591 (22.9)	672 (12.9)		790 (8.9)	63 (4.0)	218 (8.1)
Missing	6248	202	3523		4450	707	1144
Adult SEP							
Education							
Low	9920 (72.1)	4919 (46.1)	1122 (12.8)	3081 (75.3)	4944 (50.3)	1394 (78.9)	1582 (65.8)
Middle	3542 (25.8)	4463 (41.8)	5150 (59.0)	708 (17.3)	4295 (43.7)	193 (10.9)	660 (27.4)
High	289 0 (2.1)	1299 (12.2)	2464 (28.2)	302 0 (7.4)	600 0 (6.1)	181 (10.2)	163 0 (6.8)
Missing	16	841	11	311	3528	543	1435
Wealth							
Q1	2992 (23.0)	2211 (19.5)	NA	1252 (35.1)	2665 (20.0)	497 (21.5)	729 (19.1)
Q2	2610 (20.1)	2244 (19.8)		956 (26.8)	2646 (19.9)	490 (21.2)	753 (19.7)
Q3	2565 (19.7)	2291 (20.2)		533 (15.0)	2688 (20.2)	416 (18.0)	731 (19.1)
Q4	2409 (18.5)	2275 (20.1)		446 (12.5)	2724 (20.5)	471 (20.4)	792 (20.7)
Q5	2422 (18.6)	2303 (20.3)		376 (10.6)	2583 (19.4)	433 (18.8)	816 (21.4)
Missing	769	198		839	61	4	19
Healthy ageing score*							
Mean (SD)	48.2 (9.0)	49.0 (9.2)	48.6 (9.2)	47.5 (11.6)	48.6 (9.7)	40.8 (10.5)	44.4 (12.4)
Missing	210	2	2	322	348	26	23

* A higher score indicates better health with a range between 0 and 100.

CHARLS, China Health and Retirement Longitudinal Study; ELSA, English Longitudinal Study of Ageing; H2000/11, Health 2000–2011 Survey; HAPIEE, Health, Alcohol and Psychosocial factors in Eastern Europe Study; NA, not available; SAGE, WHO Study on Global Ageing and Adult Health; SEP, socioeconomic position.

The associations between childhood SEP and healthy ageing scores are reported in table 2 for parental occupation (CHARLS, ELSA, HAPIEE, and three countries in SAGE) and in table 3 for parental education (CHARLS, H2000/11 and three countries in SAGE). The associations between childhood SEP and healthy ageing scores were similar between men and women in the included cohorts. All country-specific estimates showed positive associations between parental occupation and healthy ageing after adjusting for age and sex. A high level of parental occupation was associated with higher healthy ageing scores by 1.18 points (95% CI 0.49, 1.86) in HAPIEE and 3.40 points (95% CI 2.80, 4.01) in SAGE—China. Adjusting for adult SEP variables of education and wealth attenuated the effect sizes of parental occupation.

**Table 2:** The associations between parental occupation and healthy ageing scores across studies

	CHARLS—China	ELSA—England	HAPIEE—Poland	SAGE—China	SAGE—Mexico	SAGE—South Africa
	Coeff. (95% CI)	Coeff. (95% CI)	Coeff. (95% CI)	Coeff. (95% CI)	Coeff. (95% CI)	Coeff. (95% CI)
**Model 1**						
Low	–	–	–	–	–	–
Middle	2.60 (1.81, 3.39)	1.57 (1.22, 1.92)	0.67 (0.03, 1.30)	2.76 (2.21, 3.31)	2.57 (1.17, 3.97)	0.53 (−0.41, 1.46)
High	2.41 (1.80, 3.01)	2.97 (2.56, 3.39)	1.18 (0.49, 1.86)	3.40 (2.80, 4.01)	2.76 (0.32, 5.20)	2.89 (1.34, 4.44)
P value	<0.001	<0.001	<0.001	<0.001	<0.001	0.005
**Model 2**						
Low	–	–	–	–	–	–
Middle	1.81 (1.01, 1.08)	1.01 (0.66, 1.35)	−0.26 (−0.91, 0.39)	1.91 (1.34, 2.48)	1.98 (0.56, 3.39)	0.16 (−0.74, 1.06)
High	1.29 (0.67, 1.91)	1.40 (0.97, 1.82)	−0.33 (−1.06, 0.40)	2.24 (1.63, 2.85)	1.49 (−0.96, 3.94)	0.68 (−0.89, 2.26)
P value	<0.001	<0.001	0.310	<0.001	0.011	0.421
**Model 3**						
Low	–	–	NA	–	–	–
Middle	2.17 (1.38, 2.96)	1.24 (0.89, 1.58)		1.85 (1.30, 2.39)	2.30 (0.90, 3.71)	0.25 (−0.66, 1.16)
High	1.95 (1.36, 2.54)	2.08 (1.67, 2.49)		2.36 (1.76, 2.96)	2.28 (−0.17, 4.73)	1.72 (0.17, 3.27)
P value	<0.001	<0.001		<0.001	0.001	0.061
**Model 4**						
Low	–	–	NA	–	–	–
Middle	1.58 (0.78, 2.39)	0.88 (0.53, 1.22)		1.39 (0.84, 1.95)	1.85 (0.43, 3.27)	0.17 (−0.74, 1.07)
High	1.10 (0.49, 1.71)	1.08 (0.66, 1.50)		1.70 (1.10, 2.30)	1.29 (−1.18, 3.75)	0.54 (−1.04, 2.13)
P value	<0.001	<0.001		<0.001	0.021	0.502

Model 1: adjusted for age and sex; model 2: adjusted for age, sex, education; model 3: adjusted for age, sex, wealth; model 4: adjusted for age, sex, education, and wealth.

CHARLS, China Health and Retirement Longitudinal Study; ELSA, English Longitudinal Study of Ageing; HAPIEE, Health, Alcohol and Psychosocial factors in Eastern Europe Study; NA, not available; SAGE, WHO Study on Global Ageing and Adult Health.

**Table 3:** The associations between parental education and healthy ageing scores across studies

	CHARLS—China	H2000/11—Finland	SAGE—China	SAGE—Mexico	SAGE—South Africa
	Coeff. (95% CI)	Coeff. (95% CI)	Coeff. (95% CI)	Coeff. (95% CI)	Coeff. (95% CI)
**Model 1**					
Low	–	–	–	–	–
Middle	2.53 (1.61, 3.45)	2.41 (1.63, 3.19)	3.05 (2.52, 3.59)	5.22 (2.67, 7.76)	2.38 (0.98, 3.78)
High	3.68 (1.64, 5.72)	3.22 (2.03, 4.41)	4.51 (3.31, 5.70)	2.87 (0.46, 5.29)	4.87 (2.61, 7.14)
P value	<0.001	<0.001	<0.001	<0.001	<0.001
**Model 2**					
Low	–	–	–	–	–
Middle	1.44 (0.52, 2.36)	1.52 (0.71, 2.32)	1.70 (1.16, 2.24)	3.99 (1.39, 6.60)	0.63 (–0.84, 2.10)
High	1.32 (–0.74, 3.39)	1.09 (–0.21, 2.40)	2.49 (1.29, 2.70)	0.73 (–1.80, 3.27)	1.66 (–0.74, 4.06)
P value	0.003	0.002	<0.001	0.078	0.149
**Model 3**					
Low	–	–	–	–	–
Middle	2.16 (1.25, 3.07)	1.91 (1.13, 2.70)	1.96 (1.43, 2.48)	4.81 (2.25, 7.36)	1.53 (0.06, 3.00)
High	2.71 (0.72, 4.71)	2.17 (0.97, 3.37)	2.95 (1.79, 4.12)	2.26 (–0.16, 4.68)	3.97 (1.68, 6.27)
P value	<0.001	<0.001	<0.001	0.001	<0.001
**Model 4**					
Low	–	–	–	–	–
Middle	1.33 (0.42, 2.24)	1.38 (0.58, 2.18)	1.17 (0.64, 1.70)	3.85 (1.24, 6.46)	0.31 (–1.18, 1.81)
High	0.97 (–1.06, 3.00)	0.92 (–0.37, 2.22)	1.79 (0.61, 2.96)	0.52 (–2.01, 3.05)	1.43 (–0.98, 3.83)
P value	0.008	0.005	<0.001	0.116	0.297

Model 1: adjusted for age and sex; model 2: adjusted for age, sex, education; model 3: adjusted for age, sex, wealth; model 4: adjusted for age, sex, education, wealth.

CHARLS, China Health and Retirement Longitudinal Study; H2000/11, Health 2000–2011 Survey; SAGE, WHO Study on Global Ageing and Adult Health.

Similar patterns were found for parental education (table 3). The age-adjusted and sex-adjusted results show that a high level of parental education was associated with higher healthy ageing scores by 3.22 points (95% CI 2.03, 4.41) in H2000/11 and 4.87 points (95% CI 2.16, 7.14) in SAGE—South Africa. The increasing trend from low to high levels was not found in SAGE—Mexico but the sample sizes of high/middle levels were small (<6%). Compared with low parental education, middle level was associated with 5.22 points increase in healthy ageing (95% CI 2.67, 7.76) while the difference between low and high levels was 2.87 (95% CI 0.46, 5.29). After adjusting for adult SEP variables of education and wealth, the effect sizes of parental education were attenuated. The associations between childhood SEP and healthy ageing were similar in men and women (online supplemental table S4).

The results of causal mediation analysis are reported in table 4, including estimates for the direct effects of childhood SEP on healthy ageing scores, the indirect effects via adult SEP and their total effects. The indirect effects of education were estimated to be between 29.2% (SAGE—Mexico) and 77.7% (SAGE—South Africa). For wealth, the minimum and maximum percentages were also found in SAGE—Mexico (20.6%) and South Africa (44.5%) cohorts. For the associations between parental occupation and healthy ageing scores, the mediation effects of education ranged between 29.3% (SAGE—China) and 78.7% (HAPIEE—Poland). The percentages of the indirect effects via wealth were estimated to be between 8.9% (SAGE—Mexico) and 53.2% (SAGE—South Africa). The mediation effects of education were generally stronger than those of wealth. Online supplemental table S5, supporting information, shows the results further adjusting for education or wealth and the proportion of indirect effects via adult SEP was generally similar to the main results (table 4). In online supplemental table S6, supporting information, the associations between childhood SEP, adult SEP and healthy ageing scores are reported based on the hypothesised pathways in figure 1. The direct effects (C1 and C2 in online supplemental table S6) showed the associations between childhood SEP and healthy ageing scores when including all the available SEP measures, age and sex altogether in one model. After accounting for the mediation effects of both education and wealth, the direct effects were generally small with unclear trends.

**Table 4:** Causal mediation analysis of the associations between healthy ageing scores, childhood and adulthood socioeconomic position (adjusted for age and sex)

	CHARLS—China	ELSA—England	HAPIEE—Poland	H2000/11—Finland	SAGE—China	SAGE—Mexico	SAGE—South Africa
	Coeff. (95% CI)	Coeff. (95% CI)	Coeff. (95% CI)	Coeff. (95% CI)	Coeff. (95% CI)	Coeff. (95% CI)	Coeff. (95% CI)
**Parental education (high/middle vs low)**							
Adult SEP mediator: education							
Direct	1.46 (0.46, 2.47)	NA	NA	1.45 (0.59, 2.30)	1.75 (1.04, 2.46)	2.88 (0.20, 5.56)	0.67 (−1.33, 2.67)
Indirect	1.25 (0.77, 1.72)			1.19 (0.73, 1.65)	1.58 (1.06, 2.09)	1.19 (−0.81, 3.18)	2.29 (0.84, 3.74)
Total	2.71 (0.97, 4.45)			2.64 (0.94, 4.33)	3.32 (2.46, 4.19)	4.06 (1.39, 6.74)	2.96 (0.48, 5.44)
% of indirect	46.0			45.1	47.4	29.2	77.7
Adult SEP mediator: wealth							
Direct	2.12 (1.19, 3.04)	NA	NA	2.10 (1.36, 2.84)	2.02 (1.45, 2.59)	3.12 (1.01, 5.23)	1.63 (−0.02, 3.27)
Indirect	0.55 (0.26, 0.84)			0.60 (0.35, 0.84)	1.24 (0.93, 1.54)	0.81 (−0.36, 1.98)	1.30 (0.29, 2.31)
Total	2.67 (1.78, 3.55)			2.70 (2.01, 3.38)	3.26 (2.76, 3.75)	3.93 (2.15, 5.72)	2.93 (1.68, 4.18)
% of indirect	20.7			22.1	38.0	20.6	44.5
**Parental occupation (high/middle vs low)**							
Adult SEP mediator: education							
Direct	1.55 (0.94, 2.15)	1.21 (0.88, 1.54)	0.19 (−0.48, 0.85)	NA	2.15 (1.65, 2.64)	1.77 (0.37, 3.17)	0.31 (−0.56, 1.19)
Indirect	1.00 (0.74, 1.25)	0.95 (0.82, 1.07)	0.69 (0.31, 1.06)		0.89 (0.65, 1.13)	0.84 (0.27, 1.41)	0.51 (0.30, 0.71)
Total	2.54 (1.16, 3.92)	2.16 (1.20, 3.11)	0.87 (0.05, 1.69)		3.93 (2.25, 3.82)	2.61 (−0.89, 6.11)	0.82 (−1.27, 2.91)
% of indirect	39.2	43.9	78.7		29.3	32.3	61.6
Adult SEP mediator: wealth							
Direct	1.95 (1.40, 2.50)	1.52 (1.19, 1.85)	NA	NA	1.90 (1.41, 2.40)	2.34 (1.02, 3.67)	0.38 (−0.52, 1.28)
Indirect	0.52 (0.34, 0.69)	0.52 (0.43, 0.60)			1.09 (0.87, 1.31)	0.23 (−0.17, 0.63)	0.43 (0.19, 0.67)
Total	2.47 (1.94, 3.00)	2.03 (1.71, 2.36)			2.99 (2.55, 3.43)	2.57 (1.33, 3.82)	0.81 (−0.05, 1.67)
% of indirect	21.0	25.3			36.4	8.9	53.2

CHARLS, China Health and Retirement Longitudinal Study; ELSA, English Longitudinal Study of Ageing; H2000/11, Health 2000–2011 Survey; HAPIEE, Health, Alcohol and Psychosocial factors in Eastern Europe Study; NA, not available; SAGE, WHO Study on Global Ageing and Adult Health; SEP, socioeconomic position.

## Discussion

Using harmonised cohort data from study populations in six countries across four continents, we found that higher levels of childhood SEP were associated with higher healthy ageing scores by up to five points, which was linked to 5% differences in mortality risk based on the previous study.^25^ The associations were found to be mediated by adult SEP (ie, the participants’ education and wealth) but the percentages of mediation effects varied across different populations. The mediation effects of education were generally stronger than those of wealth and explained approximately 30%–80% of the associations between childhood SEP and healthy ageing.

Our finding is in keeping with existing evidence that low childhood SEP is associated with worse health outcomes later in life^15^,^24^ and attenuated by adult SEP.^15 16 19 21^ Using a contemporary common measure for healthy ageing for older people across different countries, our study adds weight to this association in the context of LMICs where there is limited evidence to date. There are likely to be a number of possible underlying mechanisms for the association we identified. First, the accumulation of disadvantage model whereby adverse childhood conditions set in motion a cascade of disadvantages that accumulate through the life course.^38 39^ For example, being born into a low-income household is known to increase risks of adverse childhood experiences,^40^ psychosocial stress^41^ and exposure to pollution/environmental hazards.^42^ These, in turn, are likely to impact biological, cognitive and social development during childhood and lead to increased exposure to health risks, decreased opportunities for healthy behaviours and limited opportunities for income. The accumulation of these disadvantages may thereby account for differences in healthy ageing. Second, the developmental programming model whereby early-life experiences including in utero or infancy have a lasting impact on health.^43^ This model suggests that factors such as malnutrition or stress during critical periods can ‘programme’ the development of the body, thereby making one more susceptible to chronic diseases in later life. Third, the social capital and social support model whereby those with low childhood SEP may have limited social connections and thereby less access to related social, emotional, practical and financial support all of which are important in healthy ageing.^44^ Relatedly, they may have lower levels of trust and reciprocity, and experience greater levels of discrimination and prejudice. The possible models discussed are not mutually exclusive, and the specific mechanisms for individuals are complex and likely to interact with one another.

Our findings highlight that the associations between childhood SEP and health in later life were mediated by adult SEP. Supporting people to attain better adult educational and wealth outcomes may mitigate the negative impact of socioeconomic disadvantage in early life.^45^ Our results also show that lower childhood SEP was associated with lower adult education and wealth. People with low childhood and/or adult SEP often had greater physical and mental health needs but fewer resources to access and receive support needed.^46^ This may aggravate socioeconomic inequalities in healthy ageing. To investigate the complicated mechanisms and identify possible points of intervention, research needs to adapt a life course approach^38^ to understand health across the lifespan, emphasising the interconnections between different stages of life alongside wider social, economic and environmental factors.

Our study found potential variation between study populations from different countries. The distributions of childhood and adult SEP varied across countries and cohort studies. Despite efforts of data harmonisation, the classification of occupation and wealth was complicated and similar job titles might have different meanings across diverse settings. For example, those in the farming industry may be farm owners or tenant farmers in different settings.^47^ Life expectancies in some LMICs may be heavily affected by mortality in earlier life stages,^48^ leading to more selective samples of older participants. Despite variation in measurements and study populations, the associations between higher childhood SEP and better healthy ageing scores were generally observed across older populations from different countries. However, the mediation effects via adult SEP varied across different populations and particularly high percentages were found in the South African cohort. Among all the countries included in this study, South Africa has the shortest life expectancy and highest poverty and inequality.^49^ People who reached older age might have had higher SEP throughout childhood and adult life thus explaining the stronger mediation effects observed. Underlying mechanisms and cross-country variation should be further investigated in future research to inform tailored interventions for populations in diverse settings.

The study has a number of strengths, including a large sample size of over 50 000 participants and using harmonised data from five cohort studies across high-income, middle-income and low-income countries. A comprehensive measure for healthy ageing, including multiple dimensions of health and functioning, was used to compare results across older populations in diverse settings. Finally, we employed several methods to analyse mediation effects. We carried out generalised structural equation modelling to examine potential pathways (figure 1) and applied causal mediation analytical approaches to estimate the natural direct and indirect effects. However, there were some limitations. Since most studies in the ATHLOS consortium did not have any information on parental education or parental occupation, we were only able to include five cohort studies in this analysis. Recall bias could be a potential issue in these self-reported measures. However, a recent analysis from the Health and Retirement Study suggested a moderate reliability of retrospective measures for parental education and similar predictive validity for later-life health and wealth outcomes across prospective and retrospective measures.^50^ There was a high degree of missing and incomplete data on the variables of childhood and adult SEP measures. Multiple imputation was used to address missing data but the results might reflect the missing-not-at-random scenario (eg, people with very poor health). Despite data harmonisation, these cohort studies had different study designs and measurement methods. It was difficult to completely remove variation in harmonised variables across datasets—as aforementioned, for example, occupational roles implicate different social and economic status and thus meaning in different societies. In seeking to understand childhood SEP, we used crude measures of parental education and occupation. Although measures for wealth and education were likely to capture material aspects (such as housing status, financial difficulties and access to insurance)^51 52^ and behavioural and psychological aspects (such as diet, use of drugs/alcohol),^53^,^55^ it was not possible to identify specific physical, psychosocial and environmental factors and their impacts on health in later life. The analyses were adjusted for age and sex but there could be other factors (eg, genetic factors) that could affect both SEP and healthy ageing and/or have interaction effects with SEP (eg, gene–environment interaction). Inheritance of genetic factors might influence SEPs in both parents and offspring (eg, educational attainment) and contribute to the intergenerational transmission of health inequalities.^56^ Structural factors such as ethnicity, social welfare, education and healthcare systems may also modify the associations between childhood SEP and healthy ageing.^57^,^59^ For example, the social safety net and welfare policies^60 61^ can support individuals with disadvantaged backgrounds and reduce socioeconomic inequalities in health across different life stages. However, our analyses were not able to capture these differences across countries.

In conclusion, we found that high childhood SEP was associated with higher healthy ageing scores. This association varied in strength across study populations from different countries but in all cases there was evidence that it was mediated by adult education and wealth. Our findings suggest that there are opportunities through the course of childhood and in adulthood to ameliorate the negative sequelae of childhood SEP by maximising education attainment and financial security. Improvement of SEP in one generation may have beneficial influences on their offspring and future generations. Further research using a life-course approach is needed to understand and identify underlying mechanisms that explain the variations across different contexts. More longitudinal cohort studies are needed across diverse global contexts to consider a variety of mediators related to demographic, cultural, social, political and environmental factors. This will generate empirical evidence that will better inform policies and practices to address the determinants of healthy ageing and mitigate health inequalities in global ageing populations.

## Supplementary material

10.1136/bmjph-2024-001590 online supplemental file 1

## Data Availability

Data are available upon reasonable request.
