# Supplementary material for: Childhood socioeconomic position and healthy ageing: results from five harmonised cohort studies in the ATHLOS consortium
Source: BMJ Public Health. 2025 Feb 26;3(1):e001590. doi: 10.1136/bmjph-2024-001590 (PMC11865732; doi:10.1136/bmjph-2024-001590)
Supplement: online supplemental file 1 [file bmjph-3-1-s001.pdf]

**Childhood socio-economic position and healthy ageing: results from five harmonised cohort studies in the ATHLOS consortium**

**Supporting Information**

**Table S1.** Domains and items included in the ATHLOS healthy ageing score

| Cognition | Vitality | Sensory functions | Locomotion/mobility | Activities of daily living (ADL) | Instrumental activities of daily living (IADL) |
|---|---|---|---|---|---|
| • Memory<br>• Immediate recall<br>• Delayed recall<br>• Verbal fluency<br>• Orientation in time<br>• Processing speed | • Experiences some degree of pain<br>• Having high level of energy<br>• Urinary incontinence<br>• Sleeping | • Near vision<br>• Far vision<br>• Eyesight using glasses or lens as usual<br>• Hearing in general<br>• Hearing in a conversation | • Stooping, kneeling or crouching<br>• Lifting or carrying weights<br>• Climbing stairs<br>• Getting up from sitting down<br>• Walking by yourself and without any equipment<br>• Pulling or pushing large objects<br>• Sitting for long periods<br>• Reaching or extending arms<br>• Walking speed<br>• Dizziness when walking on a level surface<br>• Picking up things with fingers | • Getting in or out of bed<br>• Bathing or showering<br>• Getting dressed<br>• Moving around the home<br>• Using the toilet<br>• Eating | • Doing housework<br>• Shopping for groceries<br>• Getting out of the house<br>• Difficulties in preparing meals<br>• Using a map<br>• Managing money, bills or expenses<br>• Taking medications<br>• Making telephone calls |

**Table S2.** Classification of Childhood parental occupation

| | CHARLS | ELSA | HAPIEE | SAGE |
|---|---|---|---|---|
| High | Managers, Professionals and technicians | Manager or senior official in someone else's business, running their own business, professional or technical | Managers, Professional | Legislators, senior officials and managers, Professionals |
| Middle | Clerks, Commercial and service workers | Armed forces, administrative, clerical or secretarial, skilled trade, caring leisure, travel personal services, Sales or customer service | Technicians and associate professionals, Clerical support workers, Service and sales workers, Armed forces occupations | Technicians and associate professionals, Clerks, Service workers and shop and market sales workers, Armed forces |
| Low | Agricultural, forestry, husbandry and fishery producers, production and transportation workers | Plant, process or machine drivers or operators, Other jobs, Something else, Casual jobs, Unemployed, Sick / disabled | Skilled agricultural, forestry and fishery workers, Craft and related trades workers, Plant and machine operators, and assemblers, Elementary occupations | Skilled agricultural and fishery workers, Craft and related trades workers, Plant and machine operators and assemblers, Elementary occupations |

**Table S3.** Classification of Adult socioeconomic position

| | CHARLS | ELSA | HAPIEE | H2000/11 | SAGE |
|---|---|---|---|---|---|
| Adult education: Highest level of formal education achieved | | | | | |
| High | Less than primary or primary | Less than primary or primary | Less than primary or primary | Less than primary or primary | Less than primary or primary |
| Middle | Secondary | Secondary | Secondary | Secondary | Secondary |
| Low | Tertiary | Tertiary | Tertiary | Tertiary | Tertiary |
| Adult wealth: sources of income and assets normalised and divided into quintiles | | | | | |
| Measures for wealth | All the incomes and assets are asked per year | Financial derived variables | NA | Household gross incomes/month | Permanent income quintile |

**Table S4.** The associations between childhood SEP and healthy ageing scores by men and women (adjusted for age, education and wealth)

| | CHARLS- China | ELSA- England | HAPPIE- Poland | H2000/11- Finland | SAGE- China | SAGE - Mexico | SAGE – South Africa |
|---|---|---|---|---|---|---|---|
| | Coeff. (95% CI) | Coeff. (95% CI) | Coeff. (95% CI) | Coeff. (95% CI) | Coeff. (95% CI) | Coeff. (95% CI) | Coeff. (95% CI) |
| **Parental occupation** | | | | | | | |
| Men | | | | | | | |
| Low | - | - | - | NA | - | - | - |
| Middle | 1.73 (0.62, 2.83) | 0.89 (0.38, 1.40) | -0.69 (-1.53, 0.15) | | 1.06 (0.27, 1.85) | 2.75 (0.30, 5.19) | -0.26 (-1.69, 1.17) |
| High | 1.35 (0.52, 2.18) | 1.12 (0.48, 1.75) | -0.77 (-1.77, 0.22) | | 1.71 (0.79, 2.63) | 2.11 (-2.13, 6.34) | -0.11 (-2.41, 2.20) |
| Women | | | | | | | |
| Low | - | - | - | NA | - | - | - |
| Middle | 1.52 (0.38, 2.66) | 0.86 (0.38, 1.33) | 0.06 (-0.78, 0.89) | | 1.61 (0.84, 2.37) | 1.47 (-0.16, 3.11) | 0.36 (-0.77, 1.48) |
| High | 0.80 (-0.15, 1.75) | 1.08 (0.50, 1.65) | 0.12 (-0.89, 1.12) | | 1.63 (0.74, 2.51) | 0.76 (-2.20, 3.72) | 1.08 (-1.03, 3.20) |
| p-value* | 0.67 | 0.99 | 0.27 | | 0.56 | 0.64 | 0.68 |
| **Parental education** | | | | | | | |
| Men | | | | | | | |
| Low | - | | | - | - | - | - |
| Middle | 2.11 (0.88, 3.35) | NA | NA | 1.44 (0.33, 2.54) | 1.46 (0.68, 2.23) | 2.77 (-1.59, 7.14) | 0.38 (-1.95, 2.70) |
| High | 1.95 (-0.75, 4.66) | | | 1.34 (0.12, 2.57) | 1.37 (-0.34, 3.08) | 3.32 (-1.43, 8.08) | 2.08 (-1.52, 5.68) |
| Women | | | | | | | |
| Low | - | NA | NA | - | - | - | - |
| Middle | 0.72 (-0.55, 1.99) | | | 0.80 (-0.93, 2.53) | 0.82 (0.07, 1.57) | 4.52 (1.26, 7.78) | 0.31 (-1.54, 2.15) |
| High | -0.11 (-3.10, 2.88) | | | 1.08 (-0.86, 3.01) | 2.11 (0.48, 3.75) | -0.75 (-3.69, 2.19) | 0.69 (-2.66, 4.03) |
| p-value* | 0.19 | | | 0.97 | 0.40 | 0.29 | 0.86 |

*p-value: interaction terms between sex and childhood SEP measures

**Table S5.** Causal mediation analysis of the associations between healthy ageing scores, childhood and adulthood socioeconomic status

| | CHARLS- China | ELSA- England | HAPIEE- Poland | H2000/11- Finland | SAGE- China | SAGE - Mexico | SAGE – South Africa |
|---|---|---|---|---|---|---|---|
| | Coeff. (95% CI) | Coeff. (95% CI) | Coeff. (95% CI) | Coeff. (95% CI) | Coeff. (95% CI) | Coeff. (95% CI) | Coeff. (95% CI) |
| **Parental education (High/middle vs low)** | | | | | | | |
| *Adult SEP mediator: Education*[1] | | NA | NA | | | | |
| Direct | 1.29 (0.29, 2.30) | | | 1.12 (0.28, 1.96) | 1.15 (0.45, 1.85) | 2.51 (-0.20, 5.22) | 0.56 (-1.46, 2.59) |
| Indirect | 0.95 (0.52, 1.38) | | | 0.84 (0.42, 1.26) | 1.05 (0.58, 1.51) | 0.92 (-0.88, 2.73) | 1.91 (0.67, 3.15) |
| Total | 2.24 (1.66, 3.83) | | | 1.96 (-0.03, 3.95) | 2.20 (1.45, 2.95) | 3.44 (0.79, 6.09) | 2.47 (-0.21, 5.16) |
| % of indirect | 42.3% | | | 42.7% | 47.6% | 26.9% | 77.2% |
| *Adult SEP mediator: Wealth*[2] | | NA | NA | | | | |
| Direct | 1.47 (0.57, 2.38) | | | 1.38 (0.62, 2.14) | 1.31 (0.74, 1.89) | 2.01 (-0.15, 4.18) | 0.49 (-1.13, 2.12) |
| Indirect | 0.25 (0.06, 0.44) | | | 0.17 (0.05, 0.28) | 0.69 (0.48, 0.90) | 0.40 (-0.27, 1.06) | 0.60 (-0.04, 1.24) |
| Total | 1.73 (0.84, 2.61) | | | 1.54 (0.80, 2.29) | 2.00 (1.48, 2.52) | 2.41 (0.47, 4.36) | 1.09 (-0.27, 2.46) |
| % of indirect | 14.5% | | | 10.7% | 34.4% | 16.5% | 55.0% |
| **Parental occupation (High/middle vs low)** | | | | | | | |
| *Adult SEP mediator: Education*[1] | | | | NA | | | |
| Direct | 1.33 (0.74, 1.93) | 0.98 (0.65, 1.31) | 0.19 (-0.48, 0.85) | | 1.58 (1.09, 2.06) | 1.62 (0.20, 3.03) | 0.24 (-0.65, 1.13) |
| Indirect | 0.77 (0.54, 1.00) | 0.68 (0.56, 0.79) | 0.69 (0.31, 1.06) | | 0.50 (0.30, 0.70) | 0.59 (0.12, 1.06) | 0.27 (0.10, 0.43) |
| Total | 2.10 (0.86, 3.35) | 1.66 (0.79, 2.53) | 0.87 (0.05, 1.69) | | 2.08 (1.43, 2.73) | 2.21 (-1.01, 5.42) | 0.51 (-1.61, 2.63) |
| % of indirect | 36.6% | 40.9% | 78.7% | | 24.1% | 26.7% | 52.4% |
| *Adult SEP mediator: Wealth*[2] | | | NA | NA | | | |
| Direct | 1.33 (0.78, 1.89) | 1.01 (0.68, 1.34) | | | 1.50 (1.01, 2.00) | 1.94 (0.61, 3.27) | 0.22 (-0.67, 1.11) |
| Indirect | 0.27 (0.14, 0.39) | 0.23 (0.17, 0.29) | | | 0.66 (0.50, 0.83) | 0.06 (-0.21, 0.32) | 0.17 (-0.01, 0.36) |
| Total | 1.60 (1.05, 2.15) | 1.24 (0.91, 1.57) | | | 2.17 (1.71, 2.62) | 1.99 (0.71, 3.28) | 0.39 (-0.47, 1.26) |
| % of indirect | 16.6% | 18.4% | | | 30.5% | 2.9% | 43.7% |

[1.] Adjusted for age, sex, and wealth; [2.] Adjusted for age, sex, and education

**Table S6.** Results of the hypothesised pathways in Figure 1 (adjusted for age and sex)

| Outcomes | Exposure | CHARLS- China | SAGE- China | SAGE - Mexico | SAGE – South Africa |
|---|---|---|---|---|---|
| | | Coeff. (95% CI) | Coeff. (95% CI) | Coeff. (95% CI) | Coeff. (95% CI) |
| **Healthy ageing** | **Childhood SEP** | | | | |
| | *Education (C1)* | | | | |
| | Middle vs Low | 0.98 (0.05, 1.91) | 0.73 (0.18, 1.29) | 3.25 (0.58, 5.91) | 0.27 (-1.27, 1.81) |
| | High vs Low | 0.49 (-1.54, 2.53) | 0.99 (-0.23, 2.21) | -0.11 (-3.06, 2.83) | 1.34 (-1.34, 4.02) |
| | *Occupation (C2)* | | | | |
| | Middle vs Low | 1.51 (0.70, 2.32) | 1.28 (0.71, 1.84) | 1.59 (0.15, 3.03) | 0.16 (-0.75, 1.06) |
| | High vs Low | 0.93 (0.30, 1.56) | 1.46 (0.82, 2.09) | 1.06 (-1.81, 3.92) | 0.20 (-1.57, 1.97) |
| | **Adult SEP** | | | | |
| | *Education (A1)* | | | | |
| | Middle vs Low | 2.67 (2.32, 3.01) | 1.82 (1.47, 2.17) | 1.40 (-0.10, 2.90) | 2.28 (1.18, 3.37) |
| | High vs Low | 4.89 (3.83, 5.95) | 3.05 (2.31, 3.79) | 3.46 (1.84, 5.09) | 6.56 (4.41, 8.72) |
| | *Wealth (A2)* | | | | |
| | Q2 vs Q1 | 0.13 (-0.30, 0.57) | 1.19 (0.73, 1.64) | 1.25 (0.02, 2.48) | -0.68 (-1.89, 0.52) |
| | Q3 vs Q1 | 1.36 (0.92, 1.81) | 1.85 (1.39, 2.32) | 1.50 (0.21, 2.78) | -0.82 (-2.04, 0.40) |
| | Q4 vs Q1 | 2.21 (1.75, 2.67) | 3.02 (2.55, 3.48) | 1.71 (0.46, 2.97) | -1.40 (-2.61, -0.18) |
| | Q5 vs Q1 | 3.02 (2.56, 3.47) | 5.32 (4.83, 5.80) | 2.08 (0.77, 3.40) | 0.82 (-0.49, 2.14) |
| **Adult education** | **Childhood SEP** | | | | |
| | *Education (E1)* | | | | |
| | Middle vs Low | 0.88 (0.64, 1.11) | 1.45 (1.32, 1.59) | 1.61 (1.11, 2.11) | 1.93 (1.69, 2.17) |
| | High vs Low | 1.96 (1.37, 2.55) | 2.02 (1.73, 2.31) | 2.40 (1.80, 3.01) | 2.71 (2.24, 3.19) |
| | *Occupation (W2)* | | | | |
| | Middle vs Low | 0.92 (0.74, 1.11) | 0.78 (0.64, 0.92) | 0.71 (0.35, 1.08) | 0.17 (-0.02, 0.35) |
| | High vs Low | 1.19 (1.02, 1.36) | 0.98 (0.81, 1.15) | 0.63 (0.01, 1.25) | 0.68 (0.35, 1.01) |
| **Adult wealth** | **Childhood SEP** | | | | |
| | *Education (E2)* | | | | |
| | Middle vs Low | 0.37 (0.17, 0.57) | 0.77 (0.65, 0.88) | 0.69 (0.20, 1.17) | 1.40 (1.16, 1.64) |
| | High vs Low | 0.94 (0.48, 1.41) | 0.95 (0.69, 1.21) | 0.91 (0.39, 1.44) | 1.34 (0.90, 1.78) |
| | *Occupation (W1)* | | | | |
| | Middle vs Low | 0.47 (0.31, 0.64) | 0.63 (0.52, 0.74) | 0.42 (0.17, 0.67) | 0.47 (0.33, 0.60) |
| | High vs Low | 0.50 (0.37, 0.64) | 0.65 (0.51, 0.78) | 0.43 (-0.09, 0.96) | 0.80(0.50, 1.10) |
